# Effects of hypoxia on bronchial and alveolar epithelial cells linked to pathogenesis in chronic lung disorders

**DOI:** 10.3389/fphys.2023.1094245

**Published:** 2023-03-13

**Authors:** Rebecca Berggren-Nylund, Martin Ryde, Anna Löfdahl, Arturo Ibáñez-Fonseca, Monica Kåredal, Gunilla Westergren-Thorsson, Ellen Tufvesson, Anna-Karin Larsson-Callerfelt

**Affiliations:** ^1^ Lung Biology, Department of Experimental Medical Science, Lund University, Lund, Sweden; ^2^ Respiratory Medicine, Allergology and Palliative Medicine, Department of Clinical Sciences Lund, Lund University, Lund, Sweden; ^3^ Division of Occupational and Environmental Medicine, Lund University, Lund, Sweden

**Keywords:** BEAS-2B, hAELVi, fibrosis, growth factors, hypoxia, inflammation, lung epithelium

## Abstract

**Introduction:** Chronic lung disorders involve pathological alterations in the lung tissue with hypoxia as a consequence. Hypoxia may influence the release of inflammatory mediators and growth factors including vascular endothelial growth factor (VEGF) and prostaglandin (PG)E_2_. The aim of this work was to investigate how hypoxia affects human lung epithelial cells in combination with profibrotic stimuli and its correlation to pathogenesis.

**Methods:** Human bronchial (BEAS-2B) and alveolar (hAELVi) epithelial cells were exposed to either hypoxia (1% O_2_) or normoxia (21% O_2_) during 24 h, with or without transforming growth factor (TGF)-β1. mRNA expression of genes and proteins related to disease pathology were analysed with qPCR, ELISA or immunocytochemistry. Alterations in cell viability and metabolic activity were determined.

**Results:** In BEAS-2B and hAELVi, hypoxia significantly dowregulated genes related to fibrosis, mitochondrial stress, oxidative stress, apoptosis and inflammation whereas VEGF receptor 2 increased. Hypoxia increased the expression of Tenascin-C, whereas both hypoxia and TGF-β1 stimuli increased the release of VEGF, IL-6, IL-8 and MCP-1 in BEAS-2B. In hAELVi, hypoxia reduced the release of fibroblast growth factor, epidermal growth factor, PGE_2_, IL-6 and IL-8, whereas TGF-β1 stimulus significantly increased the release of PGE_2_ and IL-6. TGF-β1 stimulated BEAS-2B cells showed a decreased release of VEGF-A and IL-8, while TGF-β1 stimulated hAELVi cells showed a decreased release of PGE_2_ and IL-8 during hypoxia compared to normoxia. Metabolic activity was significantly increased by hypoxia in both epithelial cell types.

**Discussion:** In conclusion, our data indicate that bronchial and alveolar epithelial cells respond differently to hypoxia and profibrotic stimuli. The bronchial epithelium appears more responsive to changes in oxygen levels and remodelling processes compared to the alveoli, suggesting that hypoxia may be a driver of pathogenesis in chronic lung disorders.

## 1 Introduction

Chronic lung diseases, such as chronic obstructive pulmonary disease (COPD) and idiopathic pulmonary fibrosis (IPF), are severe lung conditions with no cures or effective treatments available. COPD is one of the leading causes of death worldwide (Quaderi and Hurst, 2018). Although the two diseases are very different in nature, a common feature is the lack of sufficient oxygen supply resulting in hypoxic milieus in the lung. In COPD, airflow obstructions and remodelling of small airways and emphysema, with destruction of alveolar capillaries and alveolar epithelial cells, result in decreased oxygen transport and alveolar hypoxia (Siafakas et al., 2007). In IPF, there is ongoing remodelling processes with excessive extracellular matrix (ECM) synthesis and deposition resulting in dense fibrotic tissue with alveolar epithelial dysfunction and hypoxia (Barratt and Millar, 2014; Senavirathna et al., 2018). Epithelial cells cover all inner surfaces and serve as a line of defence and barrier and are in direct contact with altered oxygen levels (Shukla et al., 2020; Selo et al., 2021a). The concentration of inhaled oxygen decreases from 21% throughout the respiratory tree to about 13.6% in the alveoli due to the higher water vapour and carbon dioxide concentrations in the alveoli. The bronchoepithelium is supplied with blood from the systemic circulation whereas the alveolar epithelium has an important role in respiration and is supplied by the pulmonary circulation. Hypoxic pulmonary vasoconstriction occurs in the pulmonary circulation in response to low regional partial pressure of oxygen to redirect the gas exchange to better ventilated areas thereby maintaining regional perfusion/ventilation matching in the lung. The reduced oxygen levels can occur due to bronchoconstriction or remodelling of the airways and thereby cause hypoxic conditions also in the bronchial epithelium as well as hypoxia in the alveoli. In studies with primary cells obtained from both COPD and IPF patients, epithelial cells have been seen to undergo epithelial to mesenchymal transition and induced fibrosis through a cross talk between epithelial cells and fibroblasts which have been associated with persistent airway remodelling (Sun et al., 2014; Nishioka et al., 2015; Yao et al., 2021; Mallikarjuna et al., 2022). The involvement of these cells in hypoxic condition and remodelling processes needs further attention.

A known marker of hypoxia is hypoxia inducible factor (HIF), which is a transcription factor regulating oxygen homeostasis. It regulates the expression of hypoxia-targeted genes associated with apoptosis, migration, fibrosis, inflammation and angiogenesis (Xu and Dong, 2016). Angiogenesis is promoted by hypoxia through upregulated synthesis of growth factors, such as vascular endothelial growth factor (VEGF) (Harkness et al., 2014). Hypoxia has previously mainly been studied in different cancer diseases and less in detail in ongoing remodelling processes in chronic lung diseases, highlighting the need for more research into this area. In COPD, pulmonary vascular remodelling is common and comorbidities in cardiovascular disease have negative impact on COPD prognosis (Faner et al., 2014). Lung tissue remodelling as peribronchial fibrosis, increased bronchiolar vascularisation and airway vessel thickening is frequently observed in COPD patients (Harkness et al., 2014; Mercado et al., 2015). Similar events are observed in lung tissue from patients with IPF with remodelling of pulmonary arteries, altered microvasculature and correlated to reduced lung function (Barratt and Millar, 2014; Gaikwad et al., 2022). Transforming growth factor (TGF)-β1 is a key molecule of regulating ECM production and linked to fibrosis. More recently it has been seen to regulate the expression of VEGF, inducing bronchiolar angiogenesis (Willems-Widyastuti et al., 2011). However, the effect of TGF-β on bronchial and alveolar epithelial cells during hypoxia is less known. Hypoxia has previously been shown to be involved in pathways driven by inflammation relevant for disease pathology (Bartels et al., 2013).

Hence, the aim of this study was to investigate how hypoxia and TGF-β stimuli affects the expression of cellular stress, inflammatory and remodelling markers on mRNA and protein levels in human bronchial and alveolar epithelial cells. Our findings indicate that hypoxia significantly decreased genes related to fibrosis, mitochondrial and oxidative stress, apoptosis and inflammation whereas VEGFR2 was increased in the epithelial cells. Epithelial cells responded differently to hypoxia and profibrotic stimuli with an altered release of growth factors and inflammatory mediators, where the bronchial epithelium appeared more responsive to changes in oxygen levels and remodelling processes compared to the alveoli.

## 2 Materials and methods

### 2.1 Epithelial cell cultures

The bronchial cell line BEAS-2B was cultured in RPMI 1640 Medium (Thermo Fisher Scientific, Waltham, MA, United States) supplemented with 10% Foetal Clone III serum (FCIII, HyClone Laboratories, Marlborough, MA, United States), 1% sodium pyruvate (Stock 100 mM, Sigma-Aldrich, Darmstadt, Germany) and 1% Antibiotic/Antimycotic (Thermo Fisher Scientific) at 20% O_2_, 5% CO_2_. BEAS-2B cells were used in passage 27-31. The alveolar type I cell line Cl-hAELVi was cultured according to manufacturer’s instructions (InSCREENeX, Braunschweig, Germany), where culture flasks were precoated with 2.5 mL coating solution (InSCREENeX) overnight at 37°C before seeding. Basal huAEC medium with 6% supplements (InSCREENeX) was used for culturing hAELVi at 37°C, 20% O_2_, 5% CO_2_. The hAELVi cells were used in passage 14-16.

### 2.2 Hypoxic and normoxic exposures of epithelial cells

6-well cell culture plates (Nunclon Delta Surface, Thermo Scientific) were cultured with 200,000 cells in 2 mL medium per well until 70%–80% confluence. Medium was replaced with new medium with or without addition of TGF- β1 (10 ng/mL) 10 min before exposure. Plates for hypoxia exposure were placed in a hypoxic chamber [model CO2-O2 UNIT-BL (0-20, 1-95), Okolab, Ottaviano, NA, Italy] set to 37°C, 90% humidity, 1% O_2_ and 5% CO_2_ for either 4 h or 24 h. The normoxic plates were incubated at 37°C, 21% O_2_ and 5% CO_2_ for 4 h or 24 h. To verify and the oxygen settings and validate the oxygen levels in the hypoxia chamber, the oxygen concentration was logged and measured every 30 min for 24 h using LEO (Oko-lab) to verify the oxygen settings. The apparatus operated at diffusion sampling mode and wet mode measuring the oxygen concentration of the air inside the chamber.

Post exposure, the plates were immediately put on ice and cell culture medium was collected. Selected wells for RNA extraction were washed with Phosphate Buffer Saline (PBS) and lysed with RLT buffer (Qiagen, Hilden, Germany) supplemented with 1% β -mercaptoethanol. The cell lysate was homogenized using syringes and cannulas. Wells for cell protein extraction were lysed using NP-40 lysis buffer with 1% protease inhibitor (Invitrogen, Thermo Fisher Scientific) and incubated on ice for 5 min. The collected protein lysate was centrifuged at 10,000 rpm at 4°C for 10 min and the supernatants were stored at −80°C.

For immunocytochemistry, BEAS-2B and hAELVi were cultured with 15,000 cells/well on 4-well glass Millicell EZ slides (Merck, Sigma Aldrich) at 37°C, 20% O_2_, 5% CO_2_ for 24 h. Medium was exchanged, and the slides were exposed to either normoxia or hypoxia. After 24 h, the medium was immediately aspirated, and cells were fixed with 4% formaldehyde for 15 min at room temperature (RT). Slides were washed and stored in PBS at 4°C.

### 2.3 Quantification of mRNA

The collected cell lysates for RNA were pooled within each individual experiment. The RNA purification was conducted using the Qiagen RNeasy Mini Kit (Ref 74104, Qiagen) according to the manufacturer’s instructions. RNA concentration was determined using Nanodrop 2000 Spectrophotometer (Thermo Fisher Scientific). RNA was converted into cDNA according to instructions of iScript cDNA Synthesis Kit (Bio-Rad, Cat# 1708891). cDNA samples mixed with primers in iTaq universal SYBR^®^ Green Supermix (Bio-Rad, Cat# 1725124) were added to a MicroAmp Fast 96-Well Reaction Plate (Thermo Fisher Scientific). The housekeeping genes used were Glyceraldehyde 3-phosphate dehydrogenase (GAPDH), β -actin and 18S. (See specific primers in Supplementary Table S1). An Applied Biosystems StepOnePlus Real-Time qPCR system was used to run the qPCR. During the qPCR, the samples were initiated at 95°C for 10 min. Thereafter, 45 cycles were performed where the samples were pre-denaturated at 95°C for 10 s, primer extended at 60°C for 30 s and cDNA synthesized at 74°C for 30 s. The qPCR data was presented as the ratio of hypoxia/normoxia. A geometric mean of all housekeeping genes, within each cell type and individual *in vitro* experiment, was used for normalisation. A fold change of <2 was considered a stable housekeeping gene (Supplementary Figure S1). Following formula was used:

Fold change=2^−CTtarget gene−CTmean houskeeping gene

### 2.4 Quantification of total protein amount

Protein concentration in cell lysates was determined using a Pierce bicinchoninic acid (BCA) Protein Assay Kit (Thermo Fisher Scientific) according to the manufacturer’s instructions. The absorption was measured at 562 nm with a microplate reader (Multiskan GO, Thermo Fisher Scientific) and total protein concentrations were calculated.

### 2.5 Enzyme linked immunosorbent assay

The release of VEGF-A and VEGF-C after 24 h exposure of normoxic or hypoxic condition was analysed in the cell culture medium using Human VEGF Quantikine ELISA kit DVE00 and Human VEGF-C Quantikine ELISA kit DVC00 (both from R&D Systems, Minneapolis, MN, United States), according to manufacturer’s instructions. The absorption was measured at 450 and 570 nm (as reference) with a microplate reader (Multiskan GO, Thermo Fisher Scientific). The lower detection limit of quantification was 15.6 pg/mL for VEGF-A and 55 pg/mL for VEGF-C.

The release of prostaglandin E_2_ (PGE_2_) was analysed with PGE_2_ ELISA kit from Cayman Chemical, Ann Arbor, Michigan, United States, according to manufacturer’s instructions. The absorption was measured at 450 nm with a microplate reader (Multiskan GO, Thermo Fisher Scientific). The assay detection limit for PGE_2_ was 7.8 pg/mL. The total amount of released growth factor was normalized to total protein concentration amount for each individual sample.

### 2.6 Multiplex analysis

The release of several cytokines and growth factors were analysed in cell culture medium from cells exposed to 24 h normoxic or hypoxic conditions using multiplexed Luminex discovery assays from Bio-Techne (Minneapolis, MN, United States) on a Luminex platform (Bio-Plex 200, Bio-Rad Life Science, Hercules, CA) according to the instructions given by the manufacturer. The following cytokines were analysed: monocyte chemotactic protein-1 (MCP-1), interleukin (IL)-1b, IL-6, IL-8, fibroblast growth factor-basic (FGF-basic, also known as FGF-2), hepatocyte growth factor (HGF), vascular endothelial growth factor receptor 2 (VEGFR2), VEGFR3, epidermal growth factor (EGF) and Tenascin C. The calibration curves were fitted using a five-point regression model and the results were evaluated in the Bio-Plex Manager Software 6.0 (Bio-Rad). The lower limit of detection was 35 (MCP-1), 1.5 (FGF basic), 3.6 (IL-6), 140 (VEGFR2), 9.0 (EGF), 14 (IL-1β), 3.1 (IL-8), 49 (Tenascin C), 60 (VEGFR3) pg/mL, respectively. The total amount of released mediator was normalised to total protein amount for each individual sample.

### 2.7 Immunocytochemistry staining

Immunocytochemistry staining was performed on BEAS-2B and hAELVi cells for the protein expression of HIF2 α, VEGFR2 and VEGFR3. Chamber slides were washed in tris buffered saline solution (TBS) for 5 min. Primary antibodies against HIF2 α (#NB100-132, Novus Biologicals, Bio-Techne), VEGFR2 (AHP1327, Biorad) and VEGFR3 (ab27278, Abcam, Cambridge, United Kingdom) were diluted 1:100 with dilution buffer [TBS +2% Bovine Serum Albumin (BSA)]. Omitting the primary antibody was used as negative control. Slides were incubated at RT for 90 min without light exposure and then washed in TBS for 2 min × 5 min. The HIF2 α (goat anti-mouse Alexa A647 A21240, Invitrogen; 1:200) and VEGFR2 and VEGFR3 secondary antibodies (donkey anti-rabbit Alexa flour 488 IgG A21206, Thermo Fisher Scientific; 1:200) were supplemented (separately) with 4′,6-diamidino-2-phenylindole (DAPI; 1:500) for nuclear staining. The slides were incubated at RT for 45 min without light exposure before washed with TBS 2 min × 5 min. Slides were mounted with fluorescence mounting medium (Dako) and scanned with Olympus Automatic Virtual Slide Scanner System, VS120. All the images corresponding to the same cell type and staining were adjusted for brightness using the same thresholds for appropriate comparison. The staining was quantified using the software QuPath v0.4.2 (Bankhead et al., 2017), in which a fixed threshold was set to determine the number of positive cells using the native QuPath plugin for this purpose. The signal from either the HIF2*α* or VEGFR3 secondary antibodies in the whole cell body (cytoplasm and nucleus) was considered for the quantification. Positive cells for HIF2*α* were counted at a 1000 maximum pixel intensity for BEAS-2B and 900 for hAELVi. VEGFR3 positive cells were counted at an 1800 maximum intensity for BEAS-2B and 2750 for hAELVi. At least 1000 cells (positive and negative) were counted per chamber, and a total of three to six chambers (from three-six different experiments, *n* = 3–6) per cell type and staining were used for the counting.

### 2.8 Cell viability using Lactate dehydrogenase assay

BEAS-2B and hAELVi were cultured with 7,000 cells/well in a 96-well Standard Flat base Plates (Sarstedt, Nümbrecht, Germany) and exposed to hypoxic and normoxic conditions for 24 h. A Lactate dehydrogenase (LDH) quantification was conducted using a Cytotoxicity Detection Kit (Roche, Sigma Aldrich) in accordance with the manufacturer’s instructions. 1% Triton X-100 (Sigma Aldrich) was added to some of the wells (high controls) as well as controls with only medium (low controls). The following formula was used to determine cytotoxicity:

$$Cytotoxicity=\frac{\exp.\;value-low\;control}{high\;control-low\;control}\;x\;100$$

### 2.9 Metabolic activity—Water soluble tetrazolium salt test

The cells culture plates with cells prepared for the LDH assay were further analysed for metabolic activity using a Water-soluble tetrazolium salt 1 (WST-1) test (Sigma-Aldrich). Remnants of cell medium were removed and 200 μ L of 10% WST-1 medium/well were added. Plates continued exposure to hypoxic or normoxic conditions for 1 h. Absorbance of cell culture medium was measured in a microplate reader (Multiskan GO) at 440 and 620 nm (as reference).

### 2.10 Quantification of cell amount

Cell amount was determined as previously described (Westergren-Thorsson et al., 2018). BEAS2B and hAELVi were plated 7,000 cells/well in 96-well Standard Flat base Plates (Sarstedt) overnight and then exposed to either hypoxic or normoxic condition for 24 h. Control of the plates were fixed at the start of the exposure as a comparison for cell growth and the exposed plates were fixed after 24 h. Cells were fixed in 1% glutaraldehyde (Sigma-Aldrich), stained with 0.1% crystal violet (Sigma-Aldrich) and stained with 0.1% crystal violet (Sigma-Aldrich) for 30 min. Excess staining solution was washed away and cells were permeabilised overnight with 1% Triton X100 at 4°C (Merck, Darmstadt, Germany). Changes in cell amount were quantified with a microplate reader (Multiskan GO, Thermo Fisher Scientific), measuring absorbance at 595 nm in collected cell suspension.

### 2.11 Data presentation and statistical analysis

All statistical analyses were performed using the software GraphPad Prism 9.3.1 (San Diego, United States). Four independent *in vitro* experiments were performed with two biological replicates in each experiment (*n* = 8 for each exposure), unless otherwise stated. Samples for qPCR were pooled generating 4 individual samples. The data is presented as mean with standard deviation (SD) for normoxia and hypoxia for each cell type. Student’s *t*-test was used to compare statistical significance between two groups for the LDH and WST-1 data. One-way ANOVA followed by *post-hoc* analysis with Fisher’s LSD test were used for the analysis of VEGF, PGE_2_ and multiplex data. qPCR data was analysed with One sample *t*-test where normoxia was set to 1. Statistical significance was determined at *p* < 0.05 and indicated in the figures as *p < 0.05, **p < 0.01, ***p < 0.001 and ****p < 0.0001.

## 3 Results

### 3.1 Hypoxia induced altered gene expression in epithelial cells

**FIGURE 1:**
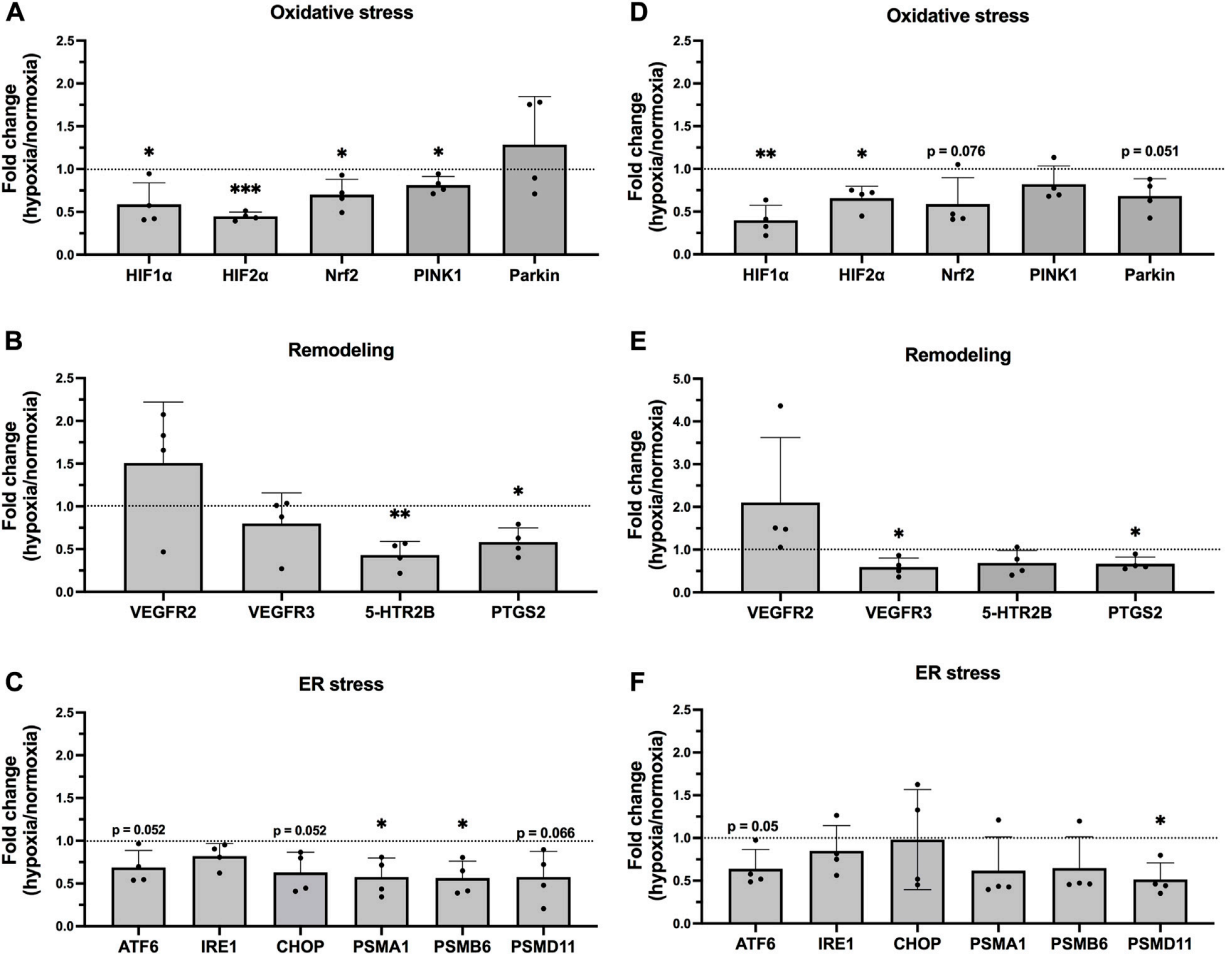
mRNA levels in epithelial cells exposed to hypoxia. Hypoxia-target genes in BEAS-2B **(A–C)** and hAELVi **(D–F)**. Data is based on four individual experiments (*n* = 4). The data is presented as the ratio of hypoxia and normoxia with mean ± SD. Black dotted line ratio = 1. Statistical analysis was performed using qPCR data was analysed with One sample *t*-test. *p < 0.05, **p < 0.01, ***p < 0.001, ****p < 0.0001.

The oxygen concentration in the air of the hypoxic chamber was confirmed as hypoxic condition verifying the oxygen settings. No large variations were observed following hypoxic or normoxic exposures in expression of the housekeeping genes in either BEAS-2B or hAELVi. Epithelial cells exposed to hypoxia for 24 h showed altered gene expression levels in comparison to normoxic exposure. Markers of hypoxia (HIF-1 α and HIF-2 α ), oxidative stress (Nrf2, PINK1 and Parkin), remodelling (VEGFR3 and PTGS2) and ER-stress (ATF6, IRE1, CHOP, PSMA1, PSMB6, and PSMD11), as well as apoptosis (Bcl2), were decreased following 24 h hypoxia exposure. VEGFR2 showed an increase, although not significant, in both bronchial and alveolar epithelial cells (Figures 1A–F; Supplementary Figure S2).

In BEAS-2B cells, HIF1 α (*p* = 0.046) and HIF2 α (*p* = 0.0002) were significantly decreased by 24 h hypoxia exposure. The expression of the oxidative stress markers Nrf2 (*p* = 0.045) and the mitochondrial marker Pink1 (*p* = 0.034) was also significantly reduced (Figure 1A), as well as PTGS2 (*p* = 0.015) and tissue remodelling factor 5-HTR2B (*p* = 0.0057), while VEGFR2 showed tendencies towards an upregulation (Figure 1B). A significant decrease was also observed for the ER stress markers PSMA1 (*p* = 0.031) and PSMB6 (*p* = 0.022) in the BEAS-2B cells (Figure 1C). Similar pattern was seen in hAELVi, as the expression of HIF1 α (*p* = 0.0064), HIF2 α (*p* = 0.016) and Parkin (*p* = 0.051) was downregulated in cells exposed to hypoxia (Figure 1D). The receptor VEGFR2 was increased, but not significant, whereas VEGFR3 (*p* = 0.032) and PTGS2 (*p* = 0.024) were reduced (Figure 1E). A downregulation was also observed for the ER stress markers ATF6 (*p* = 0.050) and PSMD11 (*p* = 0.016) (Figure 1F).

### 3.2 Expression of HIF2 α in epithelial cells after hypoxia exposure

**FIGURE 2:**
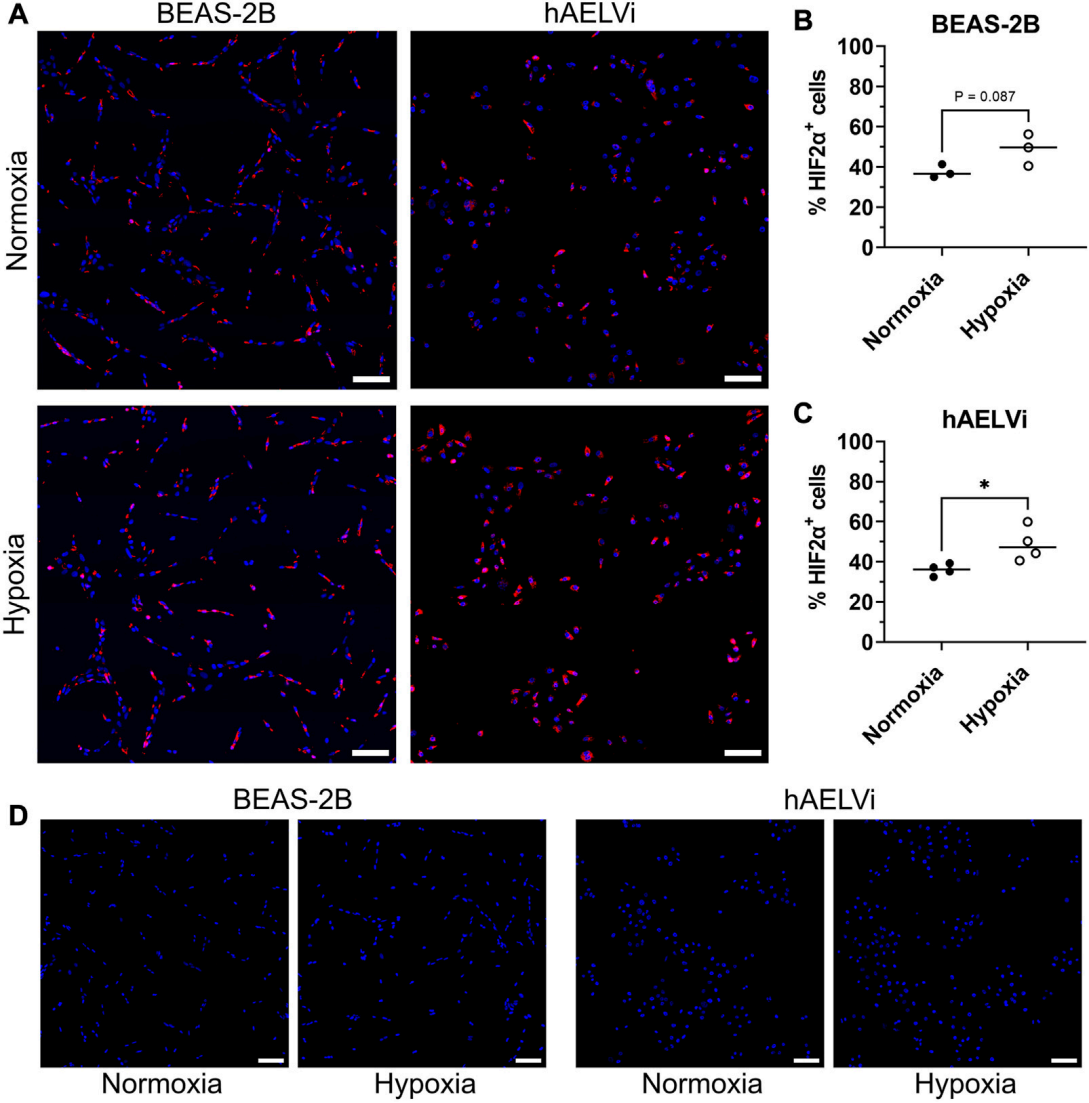
Immunofluorescence staining of HIF2 α in BEAS-2B and hAELVi exposed to normoxia compared to hypoxia **(A)**. DAPI (blue) and HIF2 α (red). Scale bar = 100 µm. Quantitative analysis of HIF2 α ^+^ cells in BEAS-2B **(B)** and in hAELVi **(C)**. Control staining without the anti-HIF2α primary antibody **(D)**. DAPI (blue) and HIF2 α (red). Scale bar = 100 µm.

To further explore the change of HIF2 α at the transcription level, the protein expression of HIF2 α was analysed by immunocytochemistry in BEAS-2B and hAELVi cells after 24 h of exposure. Comparisons were made between cells exposed to normoxia or hypoxia (Figures 2A–D). Expression of HIF2 α was detected in both normoxic and hypoxic culture conditions for both cell lines, with expression seen in both the cytoplasm and in the nucleus. Quantification of HIF2 α positive cells showed a significantly increased protein expression after hypoxia exposure in hAELVi cells (*p* = 0.029) and a trend towards increased expression in BEAS-2B (*p* = 0.087) Figures 2B, C).

### 3.3 Effect of hypoxia and profibrotic stimuli on release of growth factors

**FIGURE 3:**
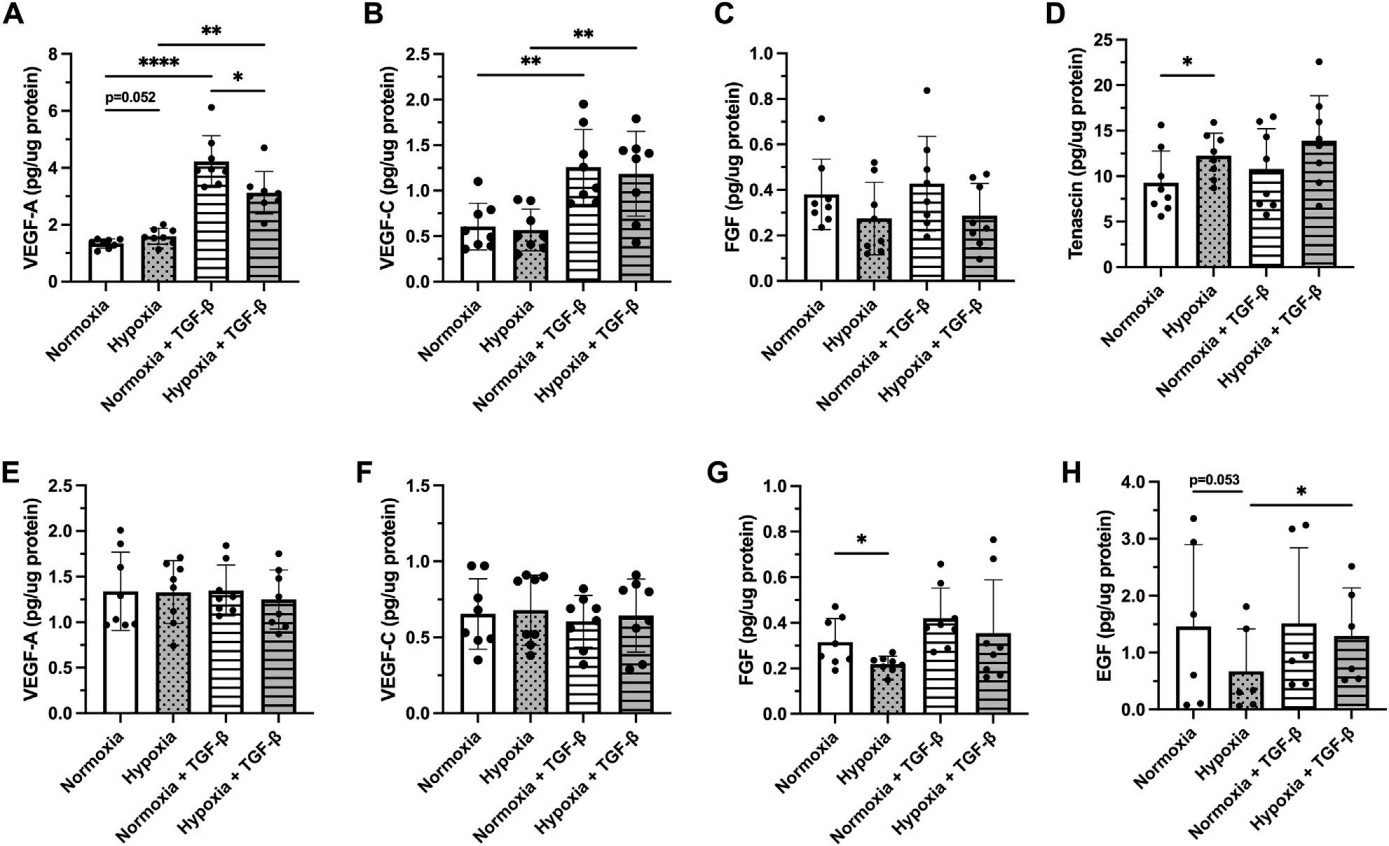
Release of following growth factors: Vascular endothelial growth factor (VEGF)-A **(A,E)** and VEGF-C **(B,F)**, fibroblast growth factor (FGF) basic **(C,G)**, Tenascin C **(D)** and epidermal growth factor [EGF, **(H)**] were investigated in BEAS-2B **(A–D)** and hAELVi **(E–H)** cells exposed 24 h to normoxia or hypoxia with or without TGF-β1 (10 ng/ml) stimuli. The total amount of released growth factor was normalized to total protein amount for each individual sample. Data is presented from four individual experiments, including two biological replicates from each experiment (*n* = 8 in total). The data is presented as mean ± SD. Statistical analysis was performed with One-way RM ANOVA followed by *post-hoc* analysis with Fisher’s LSD test. *p < 0.05, **p < 0.01, ***p < 0.001, ****p < 0.0001.

The release of VEGF-A and VEGF-C was differentially affected following treatment with hypoxia and/or TGF- β1 between bronchiolar and alveolar cells (Figure 3). The release of VEGF-A (Figure 3A) and VEGF-C (Figure 3B) was significantly increased by TGF- β in both normoxia (*p* < 0.001) and hypoxia exposed (*p* < 0.001) BEAS-2B. Hypoxia itself induced a slight increase in VEGF-A release in BEAS-2B (*p* = 0.052; Figure 3A). Cells treated with normoxia and TGF- β 1 showed more elevated levels of VEGF-A in comparison to hypoxic condition (*p* = 0.0039) (Figure 3A). The release of FGF was not significantly altered (Figure 3C), while hypoxia significantly increased the release of Tenascin C (*p* < 0.05; Figure 3D). Neither hypoxia exposure nor TGF- β1 stimuli affected the synthesis of VEGF-A (Figure 3E) or VEGF-C (Figure 3F) in hAELVi. Hypoxia exposure significantly decreased the release of FGF (*p* < 0.05; Figure 3G) and EGF (*p* = 0.053; Figure 3H), whereas the combination of hypoxia and TGF-β1 increased the release of EGF (*p* < 0.05) in hAELVi. EGF was not detected in the BEAS-2B, whereas tenascin was not detected in hAELVi. The release of HGF was not detected in either BEAS-2B or hAELVi in the multiplex analysis.

### 3.4 Expression of VEGFR3 in epithelial cells

**FIGURE 4:**
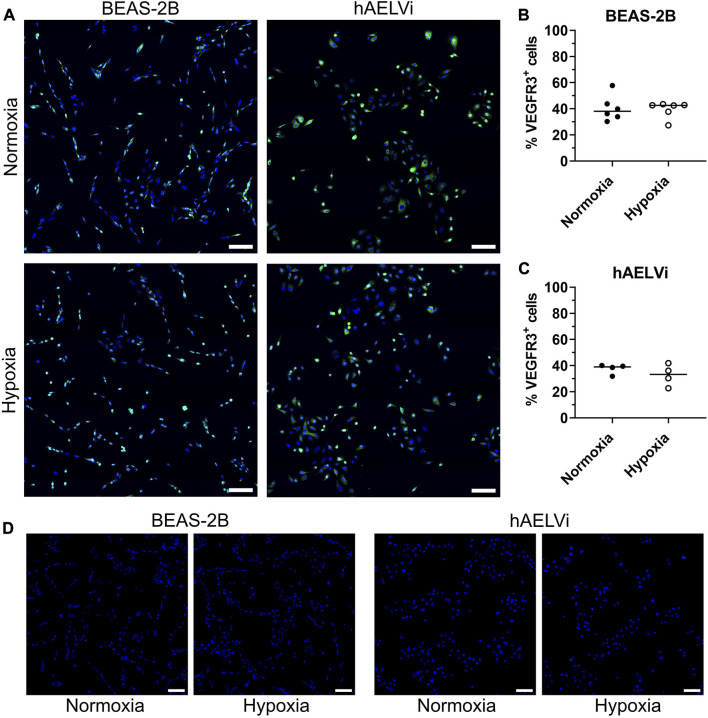
Immunofluorescence staining of VEGFR3 in BEAS-2B and hAELVi cells exposed to normoxia or hypoxia exposure **(A)**. DAPI (blue) and VEGFR3 (green). Scale bar = 100 µm. Analysis of VEGFR3^+^cells in BEAS-2B **(B)** and hAELVi **(C)**. Control staining without the anti-VEGFR3 primary antibody **(D)**. DAPI (blue) and VEGFR3 (green). Scale bar = 100 µm.

To evaluate if VEGFR expression was altered at transcriptomic level after hypoxia exposure, immunocytochemistry for VEGFR2 and VEGFR3 was performed in BEAS-2B and hAELVi cells after 24 h of exposure. Comparisons were made between cells exposed to normoxia or hypoxia. Expression of VEGFR2 was not detectable in the epithelial cells (data not shown)*.* VEGFR3 was detected in both normoxic and hypoxic culture conditions for both cell lines, with expression seen in both the cytoplasm and in the nucleus (Figures 4A–D). Quantification of number of VEGFR3 positive cells did not show a significant alteration in the expression of VEGFR3 after hypoxia exposure in BEAS-2B (Figure 4B) and hAELVi cells (Figure 4C).

### 3.5 Effect of hypoxia and profibrotic stimuli on the release of inflammatory mediators

**FIGURE 5:**
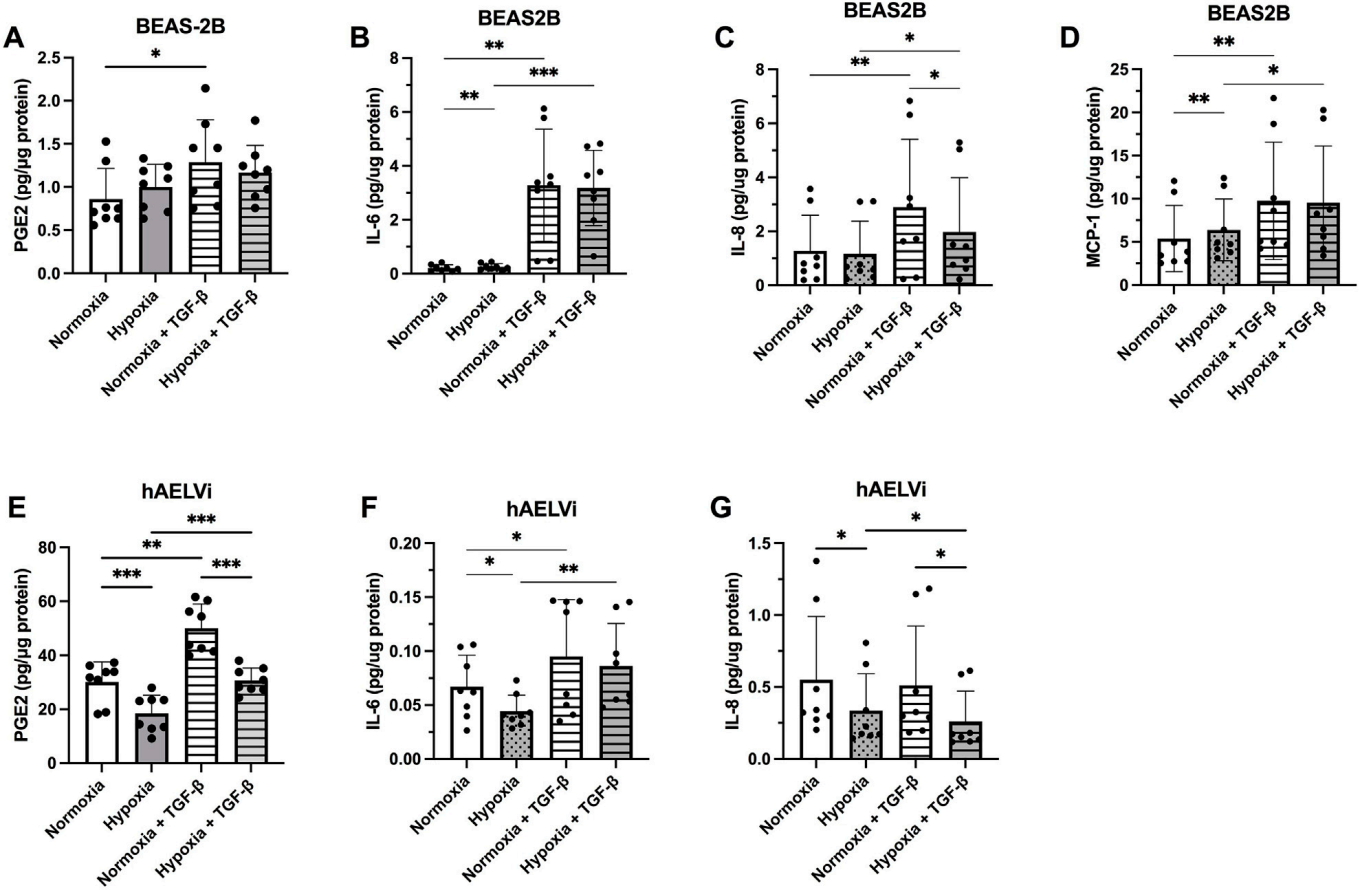
Release of the inflammatory mediators prostaglandin E_2_ (PGE_2_) **(A,E**), Interleukin (IL)-6 **(B,F**), IL-8 **(C,G)** and Monocyte chemoattractant protein (MCP)-1 **(D)** from BEAS-2B **(A–D)** and hAELVi **(E–G)** cells 24 h post hypoxia exposure, with or without 10 ng/mL of TGF- β1 stimulation. The amount of cytokines was normalized to the total protein concentration amount. All data is from four individual experiments with two biological replicates in each experiment (*n* = 8 in total). The data is presented as mean ± SD. Statistical analysis was performed using One-way ANOVA and LSD’s multiple comparison *post-hoc* test. *p < 0.05, **p < 0.01, ***p < 0.001, ****p < 0.0001.

The levels of inflammatory mediators secreted from the epithelial cells were higher in BEAS-2B for IL-6, IL-8, and MCP-1 at normoxia, compared to hAELVi. MCP-1 was not detectable in hAELVi. In contrast, hAELVi produced significantly higher amounts of PGE_2_ in normoxia, compared to BEAS-2B (Figures 5A–G). Hypoxia increased the release of IL-8 (*p* < 0.05, Figure 5B) and MCP-1 (*p* < 0.01; Figure 5D), while TGF-β1 significantly increased the release of PGE_2_ (*p* < 0.05, Figure 5A), IL-6 (*p* < 0.001; Figure 5B), IL-8 (*p* < 0.01; Figure 5C) and MCP-1 (*p* < 0.01, Figure 5D) in BEAS-2B cells. TGF-b stimulated cells though showed a decreased release of IL-8 (*p* < 0.05; Figure 5C) during hypoxia compared to normoxia.

In hAELVi, hypoxia significantly reduced the release of PGE_2_ (*p* < 0.001; Figure 5E), IL-6 (*p* < 0.05, Figure 5F), and IL-8 (*p* < 0.05; Figure 5G), whereas TGF-β1 stimulus significantly increased the release of PGE_2_ (*p* < 0.001; Figure 5E) and IL-6 (*p* < 0.05, Figure 5F). TGF-β1 stimulated cells though showed a decreased release of PGE_2_ (*p* < 0.001; Figure 5E) and IL-8 (*p* < 0.05; Figure 5G) during hypoxia compared to normoxia. MCP-1 was not detected in the hAELVi cells and the release of HGF was not detected in either BEAS-2B or hAELVi in the multiplex analysis.

### 3.6 Effect of hypoxia on cell viability, metabolic activity and cell amount

**FIGURE 6:**
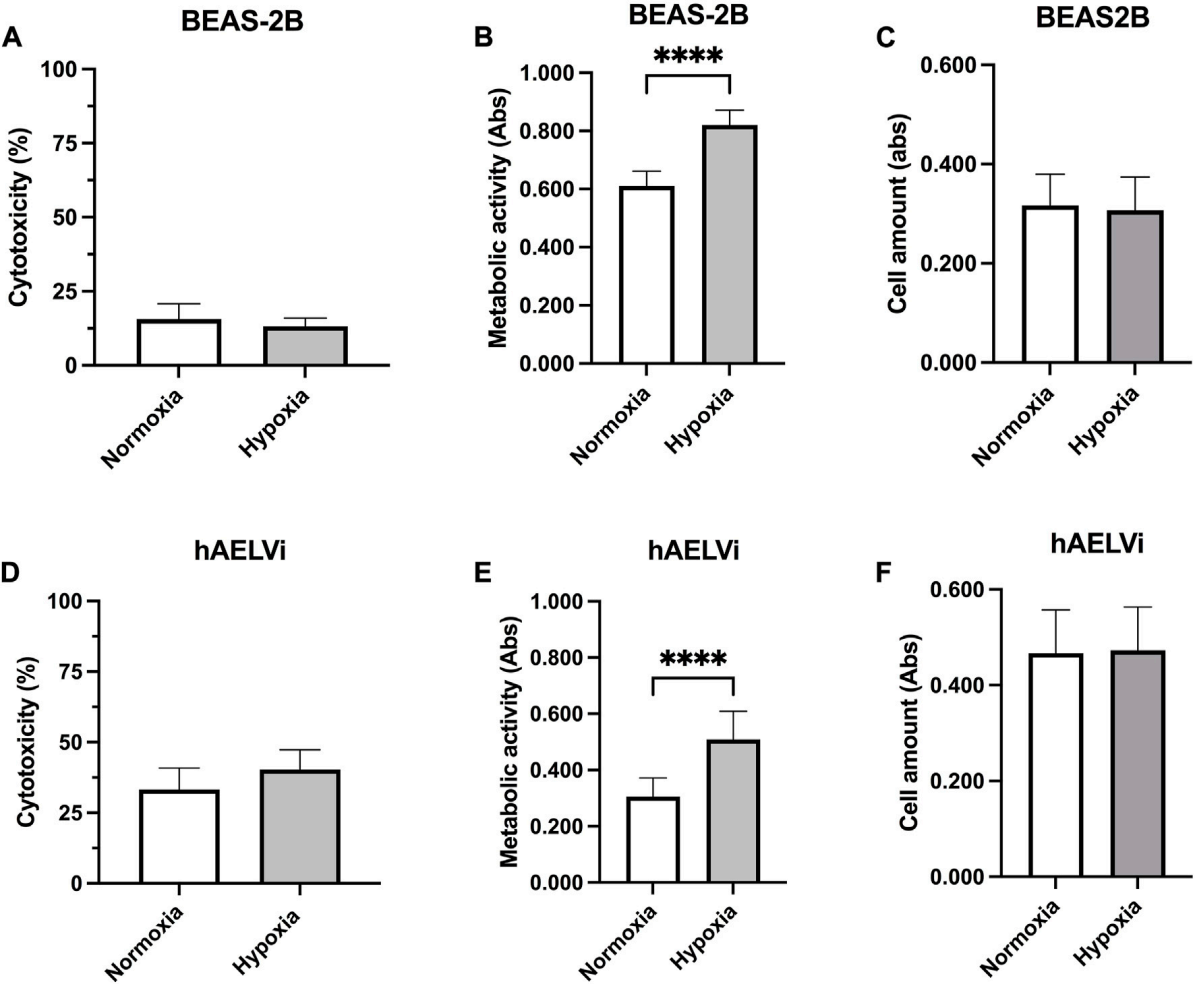
Cell viability after hypoxia exposure. Cytotoxicity measurements of BEAS-2B **(A)** and hAELVi **(D)** after 24 h exposure to either normoxia or hypoxia. Samples were related to cells treated with 1% Triton X-100, set to 100% cytotoxicity. Measured metabolic activity in BEAS-2B **(B)** and hAELVi **(E)** cells exposed to normoxia or hypoxia for 24 h. Cell amount in BEAS-2B **(C)** and hAELVi **(F)** after 24 h exposure to either normoxia or hypoxia. Data is based on 2-3 individual experiments with biological replicates (*n* = 6–8) for BEAS-2B and (*n* = 11–13) for hAELVi. Data is presented as mean ± SD. Statistical analysis was performed using Student’s *t*-test. *p < 0.05, **p < 0.01, ***p < 0.001, ****p < 0.0001.

Cell cytotoxicity was examined in epithelial cells exposed to 24 h normoxia or hypoxia, through LDH assay to examine the effect of cultured conditions (Figure 6). There were no significant differences in LDH release between hypoxic and normoxic conditions in either BEAS-2B (Figure 6A) or hAELVi cells (Figure 6D). hAELVi showed a higher release of LDH compared to BEAS-2B independently of hypoxia exposure. Metabolic activity was higher in BEAS-2B compared to hAELVi (*p* < 0.0001) during both normoxic and hypoxic conditions (Figures 6B, E). Metabolic activity was significantly increased in both BEAS-2B (*p* < 0.0001; Figure 6C) and hAELVi (*p* < 0.0001; Figure 6F) after hypoxia exposure. There were no significant changes in cell amount between normoxic and hypoxic conditions in either BEAS-2B (Figure 6E) or hAELVi (Figure 6F).

## 4 Discussion

The current study investigated how hypoxia (1% O_2_) affects human bronchial and alveolar epithelial cells and the expression of markers for angiogenesis, cellular stress, inflammation and remodelling processes. These selected markers are of importance in mechanisms involved in the pathogenesis of pulmonary diseases and may give a greater insight on the regulation of these factors during changes in oxygen levels in the lung. We found that hypoxia influenced gene expression markers associated with oxidative and cellular stress, inflammation and ECM remodelling in both BEAS-2B and hAELVi cells. Stimulation with profibrotic TGF- β1 significantly altered the release of growth factors and inflammatory mediators, a response that was altered by exposure to hypoxia in the epithelial cells.

Hypoxia is known to induce the transcription factors HIF-1α and HIF-2α by regulating the expression of hypoxia-target genes. In the current study, we observed a significant downregulation of HIF-1α and HIF-2 α at mRNA level after 24 h of hypoxia exposure, in comparison to normoxia. A similar decrease of HIF-1α stimulation during prolonged hypoxia (more than 6 h of exposure) was observed in the alveolar epithelial cell line A549 (Uchida et al., 2004) implying that gene expression levels of HIF may be swiftly and transiently induced by hypoxia at an early stage. We therefore analysed HIF-2 *α* protein expression after 24 h of hypoxia exposure using immunocytochemistry. hAELVi showed a significantly increased expression of HIF-2α after 24 h post-hypoxia, localised to cell cytoplasm and nucleus. The staining patterns align with what is known that the heterodimer of HIF α and HIF β translocate from the cytoplasm into the nucleus upon hypoxia (Xu and Dong, 2016), thus indicating a hypoxic cellular response in the studied cell culture. An integrative analysis of gene expression profile showed that downstream genes, as VEGF, regulated by HIF-2α inversely correlated with disease severity in COPD and that exposure to tobacco smoke downregulated the expression of HIF-2α (Yoo et al., 2015). In line with these findings, it was shown in a transgenic mouse model treated with the VEGFR2 inhibitor SU5416, which is known to induce emphysema, that HIF-2α plays a vital role in protecting the lung from emphysema by inducing hepatocyte growth factor, an important growth factor together with VEGF to maintain endothelial cell function and vasculature in the alveoli (Pasupneti et al., 2020). Reduced expression of HIF-2*α* in the mouse model correlated with reduced endothelial HIF-2α expression in lung tissue from patients with emphysema (Pasupneti et al., 2020). Decreased mRNA levels and expression of HIF-1α protein were observed in lung tissue from patients with severe COPD, which correlated with reduced VEGF and VEGFR2 expression, and may relate to the severity of emphysema (Yasuo et al., 2011). Altogether, long-term exposure to hypoxia and the downregulation of HIF-1α and HIF-2α may thereby contribute to and drive COPD progression and disease severity towards an emphysematous pathogenesis.

As elevated levels of HIF are associated with angiogenesis and pulmonary vascular remodelling (Harkness et al., 2014; Xu and Dong, 2016; Dai et al., 2018), we investigated if hypoxia induced expression of VEGFR2 relevant for angiogenesis and VEGF3R relevant for lymphangiogenesis. The epithelial cells expressed low amounts of VEGFR2 and VEGFR3. Expression of VEGFR2 at mRNA level was increased in both BEAS-2B and hAELVi in the current study, whereas VEGFR3 was significantly decreased in hAELVi at both mRNA and protein level after hypoxia exposure. Both BEAS-2B and hAELVi released the associated receptor ligands VEGF-A and VEGF-C, which has previously not been shown for hAELVi. VEGF-C stimulates lymphangiogenesis by binding to VEGFR3 (Schwager and Detmar, 2019) and HIF-1 α has been shown to induce upregulation of VEGF-C in cancer tumours (Morfoisse et al., 2014). BEAS-2B showed a small but significant increase of VEGF-A after hypoxia exposure, which is in line with previous studies with increased expression of VEGF and HIF-1α in lung tissue from patients with chronic bronchitis (Lee et al., 2014). A positive correlation between HIF-1α and VEGF-A and VEGFR2 expression in lung epithelium and disease severity in COPD was found in lung tissue from smoking COPD patients compared to smokers and non-smokers (Fu and Zhang, 2018). COPD pathology is recognised with peribronchial fibrosis (Hogg, 2004) and vascular remodelling of pulmonary arteries (Harkness et al., 2014) in earlier disease stages. Interestingly, the release of both VEGF-A and VEGF-C was enhanced by TGF-β1 stimulus in BEAS-2B, which was altered by hypoxia exposure. These effects on VEGF observed in this study could be linked to responses induced by hypoxia exposure of bronchial epithelial cells with increased fibrosis and remodelling processes. On the contrary, TGF-β1-treated hAELVi remained unaffected by hypoxia, highlighting a diverse responsiveness of central and distal pulmonary regions toward oxygen alterations, indicating that the alveolar regions are more adaptive to oxygen changes (Clerici and Planes, 2009).

In addition to angiogenesis, other markers related to remodelling and inflammatory processes were investigated in the context of hypoxia and profibrotic stimuli. TGF-β1 and hypoxia may act in synergistic ways by inducing each other in events involved in fibrosis and inflammation (McMahon et al., 2006; Mallikarjuna et al., 2022). Hypoxia has in general an important role in normal repair processes. Injury to the epithelium that cause low oxygen supply may induce the epithelial cells to secrete mediators to recruit inflammatory cells to the repair process and re-epithelialisation. These inflammatory cells have high metabolic demands for oxygen in the repair process. HIF-1 can influence the cellular inflammatory response by switching metabolism to glycolysis (Papandreou et al., 2006) and thereby reduce oxygen consumption. HIF-1 activation and downstream signalling induce an inflammatory response, proliferation, survival, growth factor release, and matrix synthesis in the repair process. However, if the hypoxia becomes chronic HIF-1 may accumulate resulting in pathological events as fibrosis by increased myofibroblast differentiation and excessive matrix production (Hong et al., 2014). As a synergistic effect, TGF-β1 may also induce HIF-1α *via* protein stabilisation (McMahon et al., 2006) through an increase in HIF-1α protein translation *via* the PI3K pathway and mitogen-activated protein kinase pathway (Maynard and Ohh, 2007). In the current study, secretion of tenascin C was upregulated in BEAS-2B cells following hypoxia. Tenascin C is suggested as a prognostic marker for fibrosis (Bhattacharyya et al., 2016; Bhattacharyya et al., 2022). Synovial fibroblasts showed an increased expression of tenascin C both at mRNA and protein level during hypoxic conditions (Tojyo et al., 2008). In alveolar hAELVi cells, hypoxia decreased the release of FGF and EGF in the current study. EGF is known to promote epithelial to mesenchymal transition and FGF is linked to development of fibrosis. In contrast to the bronchial epithelial cells our obtained data on release of growth factors imply that hypoxia does not promote a fibrotic event by directly targeting the alveolar epithelial cells. The inflammatory markers IL-6, IL-8 and MCP-1 are recognised as drivers in pulmonary fibrosis but also in COPD (Burgoyne et al., 2021). In the present study, TGF-β1 increased the release of IL-6, IL-8 and MCP-1 in bronchial epithelial cells and IL-6 in alveolar epithelial cells. Hypoxia increased the release of IL-6 and MCP-1 in bronchial epithelial cells whereas the release of IL-6 and IL-8 were decreased in the alveolar cells. Previous studies in endothelial cells have seen an increased release of IL-6 and IL-8 in response to hypoxia (Tamm et al., 1998). A major difference in inflammatory profile between bronchial and alveolar cells was the release of PGE_2_. hAELVi cells produced excessive amounts of PGE_2_, which was significantly increased by TGF-β1 stimulus and profoundly reduced by hypoxia exposure. In line with the reduced PGE_2_ levels, the COX-2 enzyme responsible for PGE_2_ synthesis was downregulated by hypoxia at mRNA level in both cell types in the current study. PGE_2_ has been shown to have anti-inflammatory effects in the lung (Dackor et al., 2011; Barnthaler et al., 2017) and to be reduced by hypoxia in human alveolar macrophages (Hempel et al., 1996). The high amount of PGE_2_ produced by the hAELVi cells may counterbalance other pro-inflammatory events induced by hypoxia or profibrotic stimuli, as shown by Barnthaler et al. (2017) in alveolar A549 epithelial cells and by Birrell et al. (2015) in different *in vitro* and *in vivo* models.

Patients with COPD have shown deviating expressions of genes related to ER stress, lysosomes, oxidative stress, inflammation and apoptosis when compared to healthy non-smokers (Mercado et al., 2015; Eapen et al., 2018; Weidner et al., 2018; Beghe et al., 2019; Sun et al., 2019). In the current study, hypoxia reduced expression of genes related to oxidative stress (nrf2), mitochondrial function (PINK and Parkin) and ER stress (PSMA1, PSMB6 and PSMD) in the epithelial cells, with a more pronounced effect on oxidative stress and mitochondrial dysfunction in BEAS-2B compared to hAELVi. Oxidative stress is an important mechanism in the pathogenesis of lung diseases as oxidative stress activates different signalling pathways to maintain homeostasis due to elevated levels of reactive oxygen species (ROS). Inducement of ROS causes cellular imbalances inducing ER-stress. However, disturbed mitochondrial function has been associated with unfolded protein responses in which expression of CHOP, PINK1 and Parkin usually are elevated. PINK1 is implicated in the inhibition of ROS production by recruiting Parkin to the dysfunctional mitochondria inducing mitophagy (Park et al., 2018; Kaspar et al., 2021). In this study, both cell lines had significantly higher metabolic activity in hypoxia compared to normoxia, which may be due to altered mitochondrial function. The increase in mitochondrial activity is consistent with the decrease of markers correlated to mitochondrial dysfunction (PINK1 and Parkin) and ER-stress observed at mRNA level. This data implies that hypoxia induced changes on mitochondrial activity, which could also be seen as a sign of viability, a result consistent with the unaffected apoptosis with decreased blc2 mRNA level observed for hypoxia exposed BEAS-2B and hAEVLi cells. Increased metabolic activity may indicate an increase in proliferative capacity, although no significant alterations in cell amounts were detected after 24 h in the present study, suggesting that the epithelial cells did not proliferate more in hypoxia. Increased proliferation rate has previously been observed in human lung fibroblasts and vascular smooth muscle cells after exposure to 3% O_2_ (Tamm et al., 1998) and *in vivo* exposure to hypoxia (10% O_2_) in a mice model induced proliferation of bronchial club epithelial cells (Torres-Capelli et al., 2016). Hypoxia *via* HIF-1 activation may also induce autophagy as a cell survival response to avoid cell death (Mazure and Pouyssegur, 2010). Li et al. (2022), showed in different cell lines that autophagy-dependent clearance of mitochondria decreased consumption of oxygen to preserve cell viability. Altogether, these findings imply that hypoxia is associated with altered mitochondrial function in lung epithelial cells.

A strength, but also a weakness, of these *in vitro* studies is the usage of non-carcinogenic bronchiolar and alveolar cell lines (Selo et al., 2021b). BEAS-2B shows both epithelial and mesenchymal characteristic morphology with a gene expression profile predominant of mesenchymal stem cell surface markers compared to epithelial cells (Han et al., 2020). The more recently obtained cell line hAELVi mimics the morphology and cell constitution of the alveolar lung tissue with a profile towards type 1 pneumocytes due to the tight intracellular junctions with high transepithelial electrical resistance (Kletting et al., 2018). In the current study we analysed both gene expression and protein levels after 24 h. An increase or decrease in gene expression could have appeared earlier that we were not able to detect due to unstable or degraded mRNA or the opposite that the cells have to be exposed during a longer time period. However, *in vitro* studies with primary epithelial cells at different time points of exposure followed up with transcriptomic and proteomic profiling analyses are necessary for verification of the results and inclusion of histological analysis of lung material from patients with chronic lung disorders to further understand how hypoxia and TGF-β1 affect pathological events.

## 5 Conclusion

The results from this study show that hypoxia alters the expression of several crucial mechanisms involved in pulmonary diseases by downregulating markers involved in oxidative stress and mitochondrial dysfunction, altered inflammatory response and tissue remodelling in both alveolar and bronchial cells. The response to profibrotic stimuli was altered in the presence of hypoxia with more pronounced effects in the bronchial epithelium, indicating that the bronchial epithelium is more responsive to changes in oxygen levels and remodelling processes compared to the alveoli. One reason of this differences in response to hypoxia could be the high amount of protective PGE_2_ that is produced by the alveolar epithelium, another that the bronchial epithelium is responding faster to alterations in O_2_ to maintain homeostasis whereas the alveoli is more adaptive. By mimicking the hypoxic response in a controlled *in vitro* setting pathological mechanisms can be investigated and linked to disease onset and progression.

## Data Availability

The original contributions presented in the study are included in the article/Supplementary Material, further inquiries can be directed to the corresponding author.
