# Supplementary material for: Effects of hypoxia on bronchial and alveolar epithelial cells linked to pathogenesis in chronic lung disorders
Source: Front Physiol. 2023 Mar 13;14:1094245. doi: 10.3389/fphys.2023.1094245 (PMC10040785; doi:10.3389/fphys.2023.1094245)
Supplement: Supplementary file 1 [file Table1.DOCX]

Supplementary Material

# Supplementary Figures and Tables

## Supplementary Figures

*
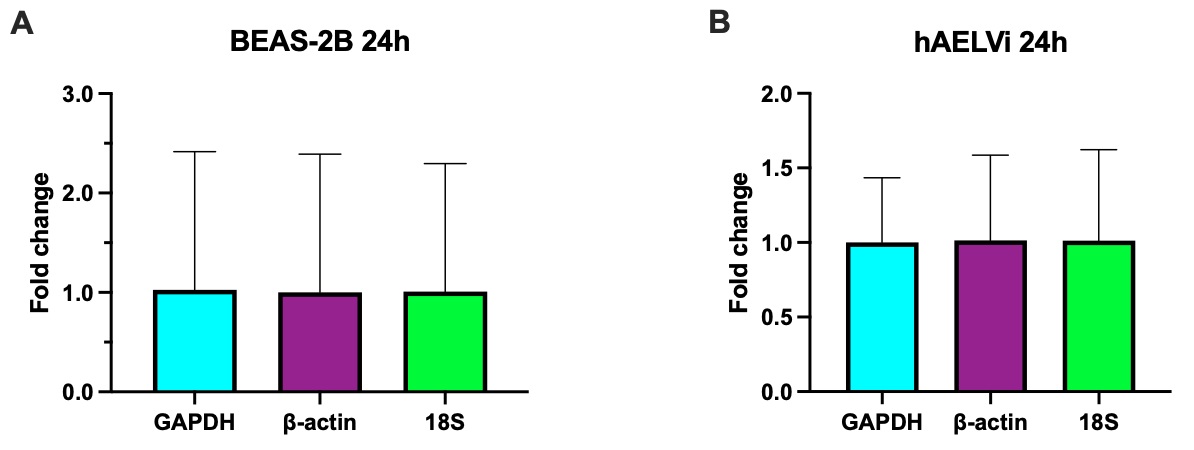
*

**Figure S1:** Analysis of housekeeping genes in qPCR data presented as fold change with mean $\pm$SD. Different cell types and cell passages were taken into consideration. The housekeeping genes were all stable for BEAS-2B (**A**) and hAELVi (**B**). Based on these results, the qPCR data of BEAS-2B and hAELVi were related to the geometric mean of all the three housekeeping genes.

**

**Figure S2:** mRNA analysis in BEAS-2B (A) and hAELVi (B) cells exposed to 24 h normoxia or hypoxia. All data is from four individual experiments (n=4). The data is presented as the ratio of hypoxia and normoxia with mean $\pm$SD. Red line indicates ratio = 1. Statistical analysis was performed using One-way ANOVA and Dunnett’s multiple comparison post hoc test ***** p < 0.05, ****** p < 0.01, ******* p< 0.001, ******** p < 0.0001.
